# Aspirin Attenuates Cardiac Allograft Rejection by Inhibiting the Maturation of Dendritic Cells *via* the NF-κB Signaling Pathway

**DOI:** 10.3389/fphar.2021.706748

**Published:** 2021-08-16

**Authors:** Xi Zhang, Aie Chang, Yanqiang Zou, Heng Xu, Jikai Cui, Zhang Chen, Yuan Li, Yifan Du, Jie Wu, Jizhang Yu, Xinling Du

**Affiliations:** ^1^ Department of Cardiovascular Surgery, Union Hospital, Tongji Medical College, Huazhong University of Science and Technology, Wuhan, China; ^2^ Institute of Hematology, Union Hospital, Tongji Medical College, Huazhong University of Science and Technology, Wuhan, China

**Keywords:** aspirin, heart transplantation, dendritic cells, allograft rejection, NF-κB

## Abstract

**Background:** Dendritic cells (DCs) serve as an important part of the immune system and play a dual role in immune response. Mature DCs can initiate immune response, while immature or semi-mature DCs induce immune hyporesponsiveness or tolerance. Previous studies have shown that aspirin can effectively inhibit the maturation of DCs. However, the protective effect of aspirin on acute cardiac allograft rejection has not been studied. The aim of this study was to elucidate the effect of aspirin exert on allograft rejection.

**Methods:** The model of MHC-mismatched (BALB/c to B6 mice) heterotopic heart transplantation was established and administered intraperitoneal injection with aspirin. The severity of allograft rejection, transcriptional levels of cytokines, and characteristics of immune cells were assessed. Bone marrow-derived dendritic cells (BMDCs) were generated with or without aspirin. The function of DCs was determined *via* mixed lymphocyte reaction (MLR). The signaling pathway of DCs was detected by Western blotting.

**Results:** Aspirin significantly prolonged the survival of cardiac allograft in mouse, inhibited the production of pro-inflammatory cytokines and the differentiation of effector T cells (Th1 and Th17), as well as promoted the regulatory T cells (Treg). The maturation of DCs in the spleen was obviously suppressed with aspirin treatment. *In vitro*, aspirin decreased the activation of NF-κB signaling of DCs, as well as impeded MHCII and co-stimulatory molecules (CD80, CD86, and CD40) expression on DCs. Moreover, both the pro-inflammatory cytokines and function of DCs were suppressed by aspirin.

**Conclusion:** Aspirin inhibits the maturation of DCs through the NF-κB signaling pathway and attenuates acute cardiac allograft rejection.

## Introduction

With the tremendous advance in the field of organ transplantation over the past half century, heart transplantation has become the standard therapy for end-stage heart failure (Stehlik et al., 2018), and the much-improved immunosuppressive agents effectively prolong the survival of patients after transplantation (Chen et al., 2020). However, the mortality associated with acute allograft rejection and subclinical episodes concerning allograft dysfunction still seriously affected the long-term survival of patients (Benza et al., 2009). Additionally, the immunosuppressive state caused by medication greatly increased the risk of cancers and infections. Side effects of pharmacotherapy such as renal toxicity and neurotoxicity are also long-term treatment barriers for transplant patients (Goldraich et al., 2020).

DCs as the potent antigen-presenting cells (APCs) derived from the bone marrow, which in response to signals or antigens can initiate both innate and adaptive immunity through natural killer (NK) cells and T cells (Reis e Sousa, 2006), play an essential role in inducing immune response as well as tolerance (Nikolic and Roep, 2013). It has been well demonstrated that the immunostimulatory or immunoregulatory properties of DCs rely on their maturation status, phenotype, and source of origin (Iberg and Hawiger, 2020). Mature DCs with high level of co-stimulatory molecules secrete pro-inflammatory cytokines, including interleukin (IL)-12, which facilitate vigorous T helper (Th) 1 cells response. Conversely, immature DCs with low level of co-stimulatory molecules induce antigen-specific hyporesponsiveness *via* the mechanisms that trigger T-cell apoptosis (Lu et al., 1999), anergy (Nouri-Shirazi and Guinet, 2002), impeding Th2 cell differentiation (Christensen et al., 2002), and cultivating regulatory T cells (Roncarolo et al., 2001). Therefore, controlling the maturation of DCs has a great potential in regulating cardiac allograft rejection. Previous studies have well established that manipulated immature or semi-mature DCs effectively prolong the allograft survival after heart transplantation (Takenaka and Quintana, 2017; Iberg and Hawiger, 2020).

Aspirin (acetyl-salicylic acid, ASA) is the most prominent member of nonsteroidal anti-inflammatory drugs (NSAIDs) for the treatment of inflammation, pain, and fever *via* the interference of cyclooxygenase (COX) II and effectively inhibits prostaglandin (PG) synthesis (Flower, 2003). Moreover, aspirin can inhibit the production of COX I and reduce the synthesis of thromboxanes (Caughey et al., 2001), which provide the pharmacological basis for the application of aspirin in reducing the risk of cardiovascular disease (Gaziano et al., 2018). In addition, previous studies have reported that aspirin served as the inhibitor for the maturation and activation of DCs through the COX-independent pathway and suppressed the immunostimulatory function of DCs (Hussain et al., 2012). Besides, aspirin also affects various cytokines production of DCs, causing DCs to be in the state of immune tolerance (Ho et al., 2001). Even in the presence of stimulators, the immunosuppressant function of aspirin-treated DCs was still reserved (Hackstein et al., 2001; Schroecksnadel et al., 2005).

The successful treatment of some autoimmune diseases resulting from the effect of aspirin on innate and adaptive immune responses also has been reported (Roehrich et al., 2015). However, whether aspirin can attenuate acute cardiac allograft rejection following heart transplantation in mice is unknown. The aim of this study was to elucidate the effect of aspirin on acute cardiac allograft rejection and to explore the underlying mechanism of these phenomena.

## Materials and Methods

### Animals

BALB/c (H-2d) and C57BL/6 (B6, H-2b) mice were purchased from Beijing Charles River. Eight-week-old male mice with the body weight of 20–30 g were selected. All animals were kept in special pathogen-free laboratories at Huazhong University of Science and Technology (Wuhan, China). Animal experiments were approved by the Animal Care and Use Committee of Huazhong University of Science and Technology.

### Heterotopic Heart Transplantation in Mice

Murine heart transplantation was performed following the method previously used in our laboratory (Wu et al., 2017). In short, donor hearts were harvested from the BALB/c mice, and the aorta and pulmonary artery of donors were anastomosed with the abdominal aorta and inferior vena cava of the recipient B6 mice. The cardiac allografts were monitored daily by palpation; complete cardiac arrest was considered as rejection. Aspirin was dissolved in DMSO and intraperitoneally injected into recipients at a dose of 200 mg/kg/d starting from the first day after transplantation. Mice in the control group were injected with DMSO only. In addition, the recipient mice were intraperitoneally injected with aspirin (200 mg/kg/d) and FK506 (1 mg/kg/d) as the combination regimen.

### Histologic Analyses of the Allografts and Immunohistochemical Characterization of Cellular Infiltrates

At the specified time (Day 5, Day 7, or Day 14), the grafts were collected, and paraffin embedding was used to make the tissue sections, then hematoxylin–eosin staining was performed as described previously (Wu et al., 2013). Parenchymal rejection (PR) grading of allografts was completed according to the modified scoring method of the International Society for Heart and Lung Transplantation (ISHLT) (Stewart et al., 2005). The other sections of grafts were stained with anti-CD4 (A0363; ABclonal) and anti-CD8 (A11856; ABclonal) antibodies for immunohistochemical analysis. Infiltration of inflammatory T cells in the graft was assessed by integrated optical density (IOD). Image-Pro Plus 6.0 software was adopted for data analysis.

### Quantitative Real-Time Polymerase Chain Reaction

Total RNA was extracted from allograft or spleen using Trizol reagent. cDNA was obtained by performing reverse transcription with ABScript II cDNA First Strand Synthesis Kit (RK20400; ABclonal). Real-time quantitative PCR was performed with SYBR premix Ex Taq on the Stepone Plus Real-Time PCR system. Copy number of the genome was quantified by comparative ΔΔCT. All results were normalized to glyceraldehyde-3-phosphate dehydrogenase (GAPDH) gene expression. Primers such as IL-2, IL-17, IFN-γ, TNF-α, T-bet, GATA-3, ROR-γt, Foxp3, and GAPDH were used for real-time quantitative PCR, as shown in Table 1.

**TABLE 1:** Primers for real-time quantitative PCR.

IL-17	Forward primer	TTT​AAC​TCC​CTT​GGC​GCA​AAA
	Reverse primer	CTT​TCC​CTC​CGC​ATT​GAC​AC
IL-2	Forward primer	TGA​GCA​GGA​TGG​AGA​ATT​ACA​GG
	Reverse primer	GTC​CAA​GTT​CAT​CTT​CTA​GGC​AC
IFN-γ	Forward primer	ATG​AAC​GCT​ACA​CAC​TGC​ATC
	Reverse primer	CCA​TCC​TTT​TGC​CAG​TTC​CTC
TNF-α	Forward primer	CCC​TCA​CAC​TCA​GAT​CAT​CTT​CT
	Reverse primer	GCT​ACG​ACG​TGG​GCT​ACA​G
T-bet	Forward primer	AGC​AAG​GAC​GGC​GAA​TGT​T
	Reverse primer	GGG​TGG​ACA​TAT​AAG​CGG​TTC
GATA-3	Forward primer	CTC​GGC​CAT​TCG​TAC​ATG​GAA
	Reverse primer	GGA​TAC​CTC​TGC​ACC​GTA​GC
ROR-γt	Forward primer	GAC​CCA​CAC​CTC​ACA​AAT​TGA
	Reverse primer	AGT​AGG​CCA​CAT​TAC​ACT​GCT
Foxp3	Forward primer	CCC​ATC​CCC​AGG​AGT​CTT​G
	Reverse primer	ACC​ATG​ACT​AGG​GGC​ACT​GTA
GAPDH	Forward primer	AGG​TCG​GTG​TGA​ACG​GAT​TTG
	Reverse primer	TGT​AGA​CCA​TGT​AGT​TGA​GGT​CA

### Generation of Bone Marrow–Derived Dendritic Cells

Dendritic cells were generated by inducing bone marrow cells from B6 mice as described previously (Son et al., 2002). Simply put, the mice were killed by neck dislocation; tibias and femurs were separated and chopped for bone marrow cells. Then cells were filtered to remove impurities. After the erythrocyte lysis was completed, the bone marrow cells were washed and resuspended with the RPMI-1640 medium (12633012; ThermoFisher) containing 10% fetal bovine serum (10099133C; ThermoFisher), 1% penicillin/streptomycin (10378016; ThermoFisher), 20 ng/ml granulocyte and macrophage colony-stimulating factor (315-03; PeproTech), and 10 ng/ml interleukin-4 (214-14; PeproTech) to the final cell concentration of 1 × 10^6^/ml. The bone marrow cell suspension was added to 96-well tissue culture plates and cultured at 200 μl per well in an incubator at 37°C in 5% CO_2_ air for 6–8 days. Every other day, half medium of the cells was replaced with the fresh one. On days 2, 4, and 6, 2 mM aspirin (HY-14654, MedChemExpress) was added or not to the medium. On the seventh day of culture, the TLR ligand, lipopolysaccharide (LPS; 100 ng/ml), was presented for 24 h to induce DC maturation.

### Enzyme-Linked Immunosorbent Assay

The serum of recipients and the culture supernatant of cells were prepared to be detected. Then target cytokines were tested by the enzyme-linked immunosorbent assay. The concentration of the target protein was calculated according to the protocol provided by the manufacturer.

### Flow Cytometric Analysis

To analyze the maturity of DCs, single suspension cells were collected and stained with Pacific Blue anti-CD11c mAb (117321; Biolegend), APC-anti-MHCII mAb (AGEL0501; Genie), FITC-anti-CD86 mAb (105109; Biolegend), PE-anti-CD80 mAb (104707; Biolegend), and PE-Cy7-anti-CD40 mAb (124621; Biolegend). Surface antigen on DCs was analyzed in CD11c+ gate. The expression levels of these antigens were indicated by the median fluorescence intensity (MFI) of positive cells. For intracellular cytokine staining, cells (1 × 10^6^) were cultured with 50 ng/ml phorbol 12-myristate 13-acetate (PMA), 0.5 mg/ml ionomycin and brefeldin for 6 h and stained with Pacific Blue anti-CD4 mAb and PerCP-CY5.5-anti-CD8 mAb for cell surface antigen. After being fixed and washed, the cells were permeabilized by 0.5% saponin and 2% FCS-PBS containing 0.1% Na-Azide. Subsequently, APC-anti-IL-17 mAb (506915; Biolegend), PE-anti-IFN-γ mAb (117321; Biolegend), and PE-CY7-anti-Foxp3 mAb (505807; Biolegend) were incubated with cells at 4°C for 30 min so that the antibodies could recognize and bind to intracellular targets. All samples were measured by flow cytometry, and FlowJO software was used for date analysis (TreeStar, Inc., Ashland, OR).

### Mixed Lymphocyte Reaction

MLR experiments were conducted in 96-well round-bottomed microculture plates. The T cells (5 × 10^5^/well) from BALB/c mice were regarded as responders. The LPS-stimulated BMDCs (mixed in a different ratio) from B6 mice incubated with 25 ug/ml mitomycin C were used as stimulators. The cell reaction was proceeded in RPMI-1640 complete medium at 37°C in 5% CO_2_ air for 3 days. T-cell proliferation was evaluated by Cell Counting Kit-8 (C0037; Beyotime). Briefly, Cell Counting Kit-8 (CCK-8) solution (10 ul) was added to each well, and cells were incubated at 37°C in 5% CO_2_ air for 3 h. The absorbance of each well was detected at 450 nm. Cell viability was determined according to the manufacturer’s instruction.

### Western Blotting

The DCs were lysed by RIPA buffer mixed with protease inhibitors. Western blotting was performed according to the method described previously (Zhang et al., 2017). Antibodies of GAPDH (AF1186; Beyotime), p65 (AF1234; Beyotime), p-p65 (AF5875; Beyotime), and IκBα (AF5204; Beyotime) were used as the primary antibodies. The horseradish peroxidase (HRP)-conjugated goat anti-rabbit IgG antibody was used as secondary antibody (A0208; Beyotime). Protein expression levels were normalized to GAPDH.

### Statistical Analysis

All data were analyzed *via* GraphPad PRISM 5.0 software. For the survival rate of allograft, Kaplan–Meier plots were constructed, and *p* values were calculated through comparing each group by log-rank. Data were expressed as mean ± standard deviation, and values were compared using the two-tailed student t test. *p* < 0.05 was considered statistically significant.

## Results

### Aspirin Prolongs the Survival of Cardiac Allograft in Mouse

**FIGURE 1:**
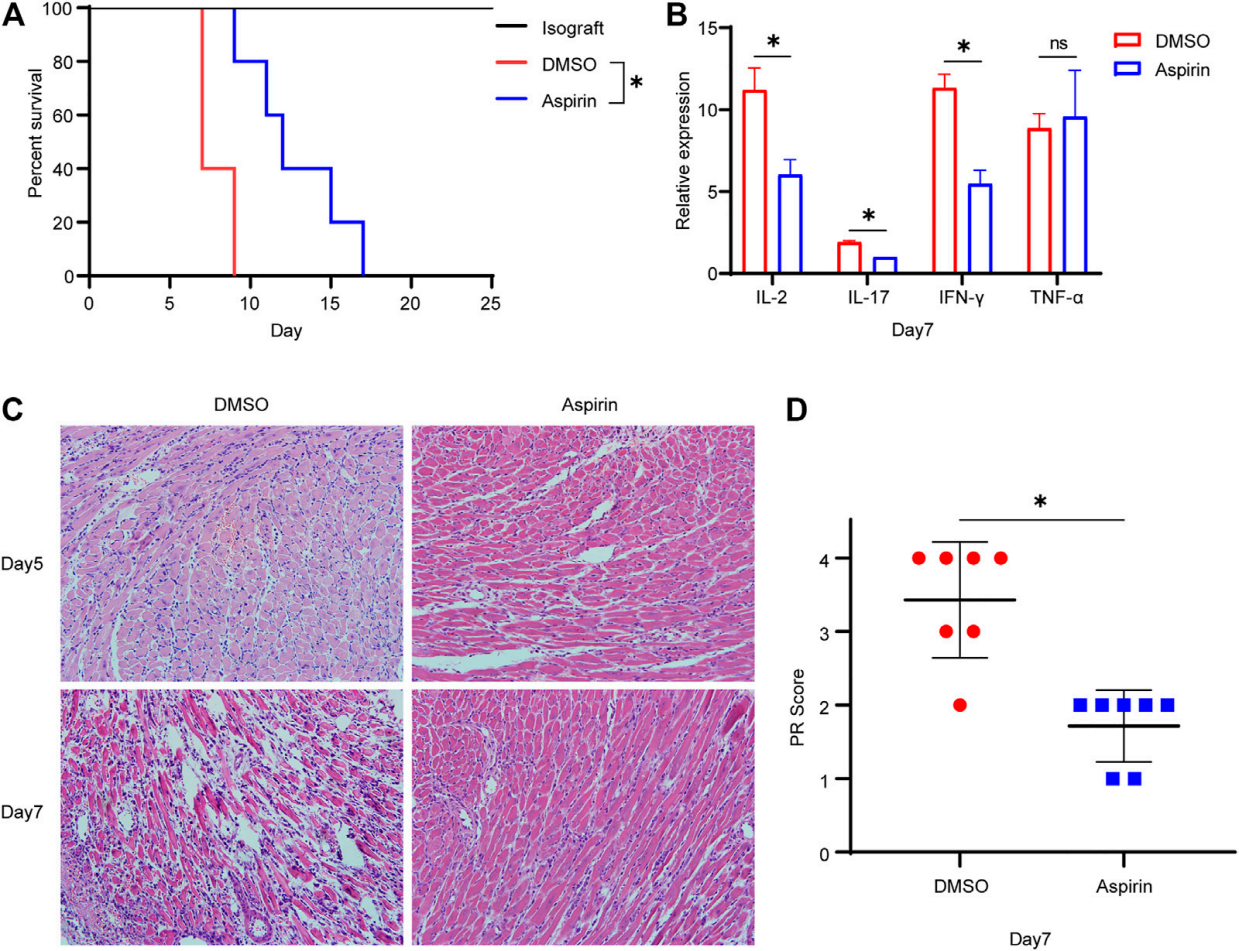
Aspirin prolongs the survival of cardiac allograft in mouse. **(A)** Establishment of the heterotopic abdominal heart transplantation model with MHC-mismatched mice (BALB/c to B6 mice). From the first day after surgery, recipient mice were intraperitoneally injected with aspirin (200 mg/kg/day). Those receiving DMSO served as control. Aspirin significantly prolonged the survival of allograft in mouse (MST, 12.8 ± 1.4 versus 7.8 ± 0.5 days, *n* = 5 per group, *p* < 0.05). **(B)** On seventh day after transplantation, total RNA was extracted from cardiac allograft and reverse-transcribed into cDNA. Transcriptional levels of IL-2, IL-17, IFN-γ, and TNF-α relative to GAPDH were detected by real-time PCR. **(C)** Cardiac allografts were harvested on the fifth and the seventh day after transplantation, tissue sections were made, and hematoxylin–eosin staining was performed (×40, *n* = 7 per group). In the aspirin-treated group, the inflammatory infiltration in the myocardial interstitium and perivascular area was significantly inhibited, and the degree of myocardial cell necrosis was reduced. **(D)** The PR score was performed on tissue sections of each group on the seventh day after transplantation (*n* = 7 per group). *p < 0.05. DMSO, dimethyl sulfoxide; MST, mean survival time; GAPDH, glyceraldehyde-3-phosphate dehydrogenase; IFN, interferon; IL, interleukin; TNF, tumor necrosis factor; PR, parenchymal rejection.

To investigate the effect of aspirin on acute rejection, we performed heterotopic abdominal heart transplantation in mice, which the hearts of BALB/c mice were considered as donors and B6 mice as recipients. Aspirin (200 mg/kg/day, intraperitoneal injection) or dimethyl sulfoxide (DMSO) was administrated from the first day after surgery. Mice treated with aspirin had a longer allograft survival time than the mice treated with DMSO (mean survival time [MST], 12.8 ± 1.4 versus 7.8 ± 0.5 days, *p* < 0.05; Figure 1A). In addition, the expression levels of interleukin (IL)-2, IL-17, and interferon (IFN)-γ in allografts from aspirin-treated mice were significantly lower than those in allografts from DMSO-treated mice (*p* < 0.05; Figure 1B). These cytokines were considered as biological markers of acute allograft rejection (Millán et al., 2014). However, the other one indicator of acute rejection, tumor necrosis factor (TNF)-α (Chang et al., 1991), showed no significant difference between the two groups (*p =* 0.71; Figure 1B). Subsequently, the effect of aspirin on acute rejection was determined by histopathologic examination; the biopsy of allograft was performed on the fifth and the seventh day after the transplantation. Hematoxylin–eosin staining showed that the inflammatory infiltration and myocardial cell necrosis of allografts were alleviated in the aspirin-treated group (Figure 1C). The parenchymal rejection (PR) score of the allograft in the aspirin-treated group was lower than that of the allograft in the DMSO-treated group (3.43 ± 0.79 versus 1.71 ± 0.49, *p* < 0.05; Figure 1D).

### Aspirin Alters the Composition of Allograft-Infiltrated T Cells and the Differentiation of T Cells in the Recipient Spleen

**FIGURE 2:**
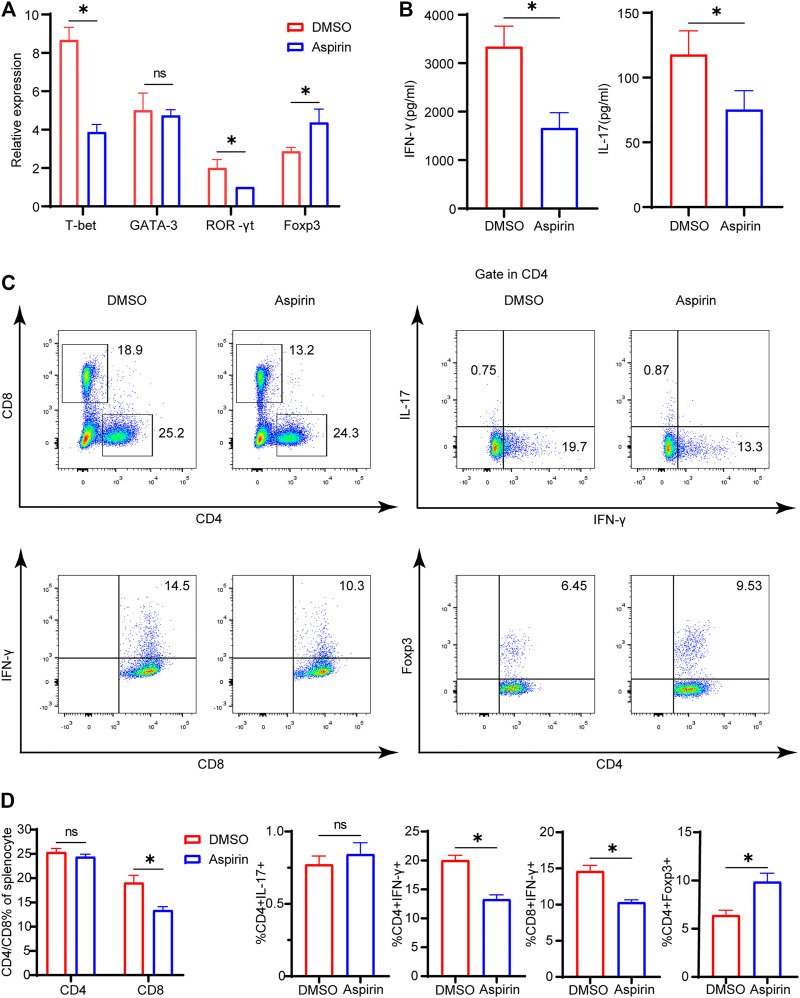
Aspirin alters the composition of allograft-infiltrated T cells and the differentiation of T cells in the recipient spleen. On the seventh day after transplantation**,** the spleens and plasma of recipient mice were collected. **(A)** The total RNA was extracted from spleens of recipient mice and reverse-transcribed into cDNA. Transcriptional levels of T-bet, GATA-3, ROR-γt, and Foxp3 relative to GAPDH were detected by real-time PCR. **(B)** The plasma of recipient mice was prepared, and the expression levels of IFN-γ and IL-17 were determined by ELISA. **(C)** The spleens of recipient mice from each group were harvested and isolated for antibody staining. The proportion of CD4(+), CD8(+), IFN-γ(+)CD4(+), IL-17(+)CD4(+), Foxp3(+) CD4(+), and IFN-γ(+)CD8(+) cells was detected by flow cytometry. The numbers represented the percentages of cells. **(D)** The percentages of CD4(+), CD8(+), IFN-γ(+) CD4(+), IL-17(+)CD4(+), Foxp3(+)CD4(+), and IFN-γ(+) CD8(+) T cells in each group. Data are presented as the mean ± SD of four independent experiments. *p < 0.05; GAPDH, glyceraldehyde-3-phosphate dehydrogenase; IL, interleukin; IFN, interferon.

On the seventh day after heart transplantation, allografts from the two groups were collected for real-time polymerase chain reaction (PCR) detection. The results showed that the specific transcription factors of Th1 cell and Th17 cell, T-bet and ROR-γt, were significantly decreased, while the Foxp3 of Treg cell was much increased in the aspirin-treated group (*p* < 0.05; Figure 2A). However, there was no statistical difference in Th2 cell transcription factor GATA-3 between the two groups (*p =* 0.65, Figure 2A). In addition, the enzyme-linked immunosorbent assay (ELISA) was performed on plasma from the two groups, and results revealed that the expression levels of IFN-γ and IL-17 in the aspirin group were decreased to different degrees compared with the DMSO group (IFN-γ, 3,343 ± 421.5 versus 1,663 ± 316.9 pg/ml, *p* < 0.05; IL-17, 117.8 ± 18.4 versus 75.3 ± 14.7 pg/ml, *p* < 0.05; Figure 2B).

Simultaneously, on the seventh day after heart transplantation, the splenocytes of recipient mice were harvested, and flow cytometry analysis was performed. We found that in the aspirin-treated group, the proportion of CD8(+) T cells in the spleen was decreased (19.1 ± 1.5 versus 13.4 ± 0.7%, *p* < 0.05; Figures 2C,D), while CD4(+) T cells did not demonstrate significant differences between aspirin and DMSO treatment (*p =* 0.65; Figures 2C,D). Subsequently, we analyzed the subsets of CD4(+) and CD8(+) T cells in the spleen and found that the proportion of IFN-γ(+)CD4(+) T cells and IFN-γ(+)CD8(+) T cells decreased, while Foxp3(+)CD4(+) T cells increased in the aspirin-treated group IFN-γ(+)CD4(+) T cells, 20.0 ± 0.9 versus 13.3 ± 0.8%, *p* < 0.05; IFN-γ(+)CD8(+) T cells 14.6 ± 0.8 versus 10.3 ± 0.4%, *p* < 0.05; Foxp3(+)CD4(+) T cells 6.4 ± 0.5 versus 9.9 ± 0.9%, *p* < 0.05; Figures 2C,D). Notably, we found the effect of aspirin and DMSO treatment on Th17 cells in the spleen did not show significant difference, which was inconsistent with their counterpart infiltrated in the cardiac allograft (*p =* 0.21; Figures 2C,D).

### Aspirin Inhibits the Maturation of DCs in the Spleen of Mouse After Heart Transplantation

**FIGURE 3:**
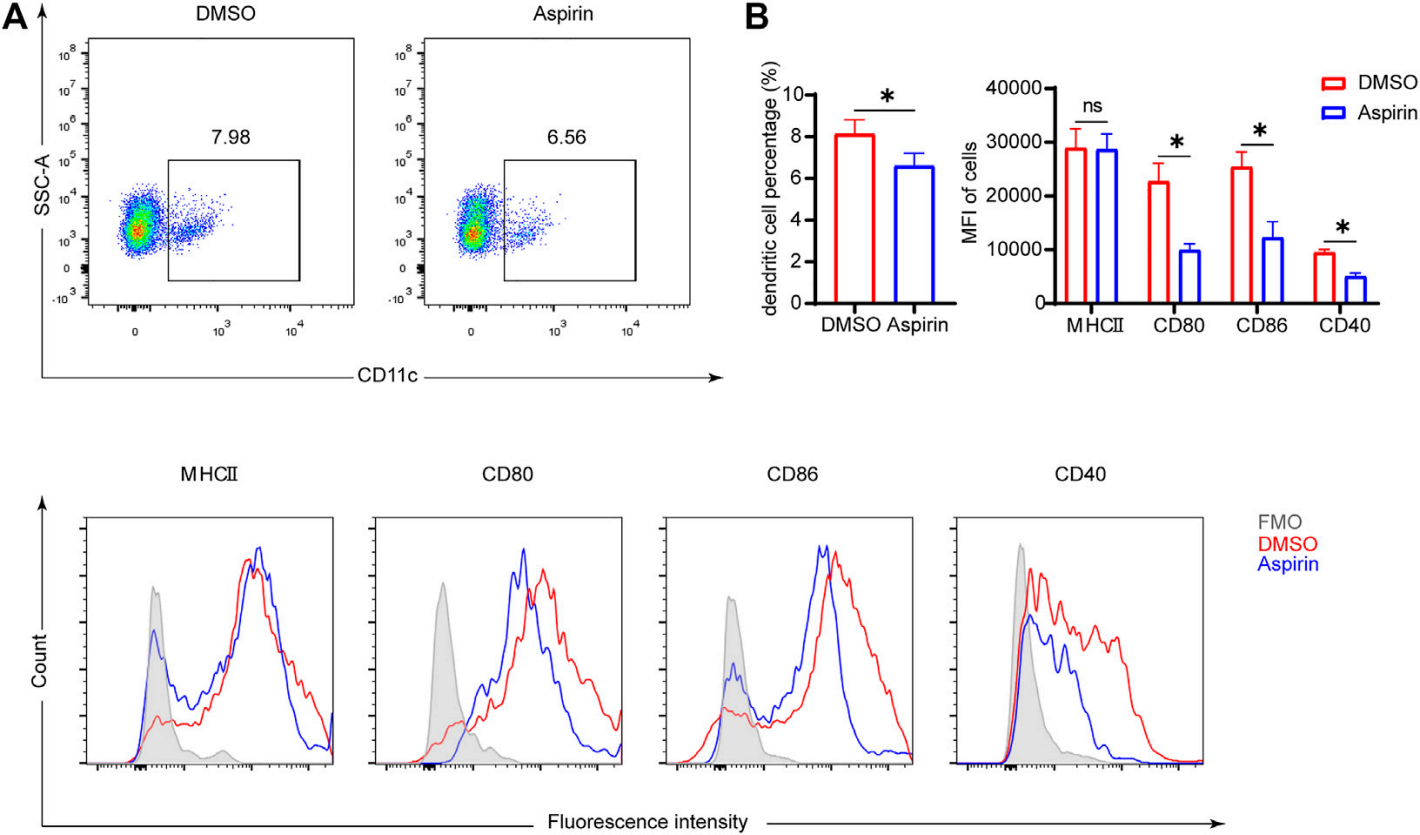
Aspirin inhibits the maturation of DCs in the spleen of mouse after heart transplantation. On the seventh day after transplantation, spleens were collected from each group and splenocytes were isolated. Fluorescence-conjugated anti-MHCII, CD80, CD86, and CD40 antibodies were used to detect the expression level of molecules on DCs. DCs were identified by anti-CD11c antibody. **(A)** The proportion of CD11c (+) dendritic cells was displayed (top), and the expression levels of MHCII, CD80, CD86, and CD40 on DCs were shown by MFI in histograms (bottom). **(B)** The percentages of DCs in spleen (left) and the expression levels of MHCII, CD80, CD86, and CD40 on DCs of each group (right). Data are presented as the mean ± SD of four independent experiments. *p < 0.05; MHC, major histocompatibility complex; MFI, mean fluorescence intensity.

As antigen-presenting cells, the maturity of DCs plays a crucial role in modulating the differentiation of naive T cells (Macri et al., 2018). The mature DCs have high expression levels of major histocompatibility complex II (MHCII), CD80, CD86, and CD40 molecules on the surface, which endorse DCs to effectively initiate the immune response (Deng et al., 2020). To investigate the effect of aspirin on DCs, 7 days after heart transplantation, we performed the cytological examination to ascertain the proportion of DCs in the spleen, and the expression levels of MHCII, CD80, CD86, and CD40 on DCs. The results showed that aspirin reduced the proportion of DCs in the spleen and effectively inhibited the expression of CD80, CD86, and CD40 on the surface of DCs (DC proportion 8.02 ± 0.68 versus 6.59 ± 0.60%, *p* < 0.05; CD80 MFI 22701 ± 3,393 versus 9,921 ± 1,205, *p* < 0.05; CD86 MFI 25413 ± 2,789 versus 12,252 ± 2,986, *p* < 0.05; CD40 MFI 9468 ± 578 versus 5,053 ± 598, *p* < 0.05; Figures 3A,B). Remarkably, after heart transplantation, there was no significant difference in the expression level of MHCII on DCs between the aspirin-treated group and the DMSO-treated group (*p =* 0.91; Figures 3A,B).

### Aspirin Inhibits the Maturation and Function of Bone Marrow–Derived Dendritic Cells *In Vitro*

**FIGURE 4:**
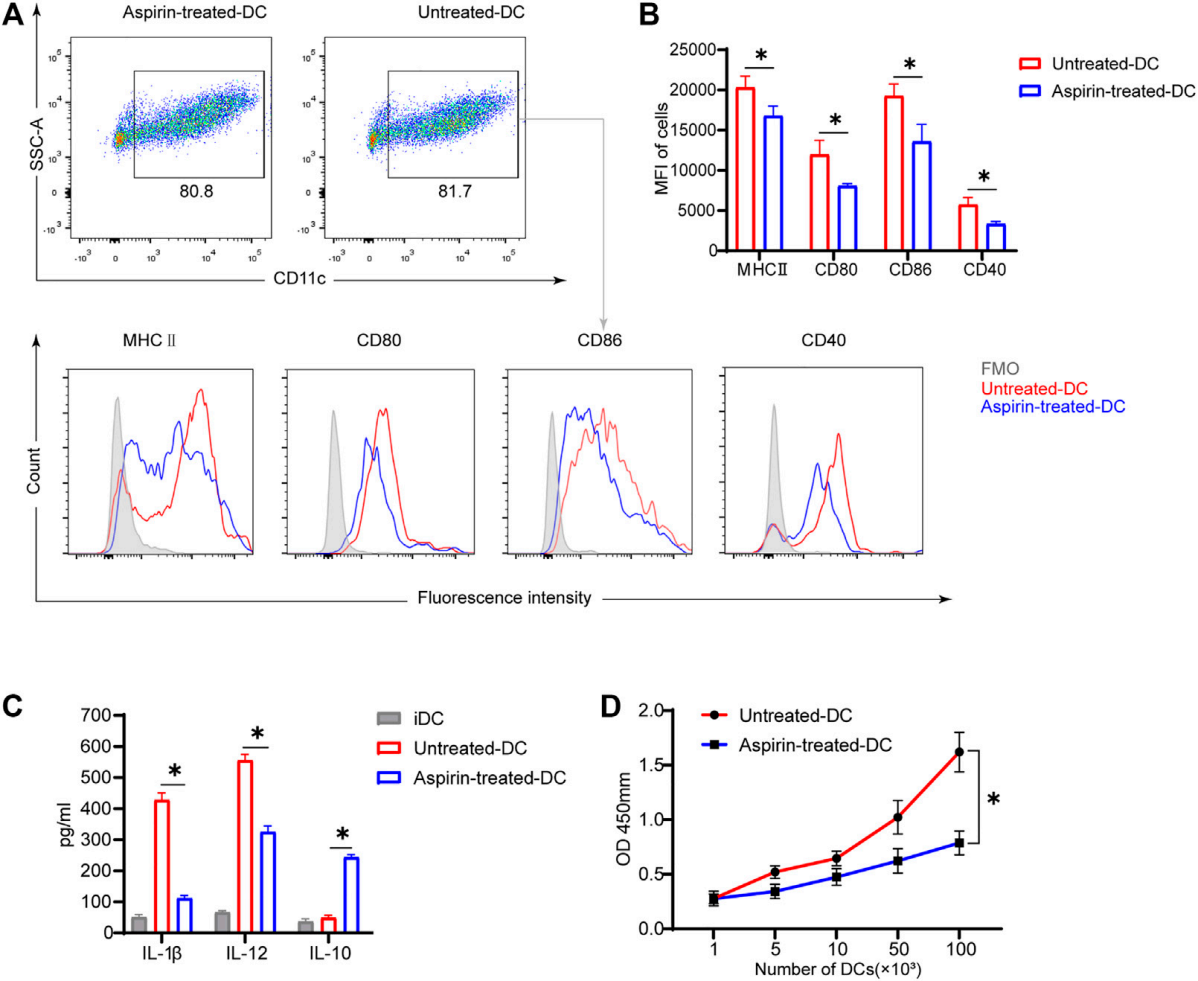
Aspirin inhibits the maturation and function of murine bone marrow–derived dendritic cells *in vitro*. BMDCs were generated from bone marrow cells (from B6 mice) with granulocyte-macrophage colony-stimulating factor (GM-CSF) and interleukin (IL)-4 induction. The aspirin-treated group was supplemented with 2 mM aspirin, while the untreated group was supplemented with DMSO only. On the seventh day of culture, cells were collected and stimulated with 100 ng/ml LPS for 24 h. After the stimulation, cells were harvested and detected by flow cytometry (the same method as Figure 3). **(A)** The proportion of CD11c (+) dendritic cells was displayed (top), and the expression levels of MHCII, CD80, CD86, and CD40 on DCs were shown by MFI in histograms (bottom). **(B)** The expression levels of MHCII, CD80, CD86, and CD40 on DCs of each group. **(C)** After stimulation of DCs, the supernatant of medium in each group was collected, and the levels of IL-1β, IL-12, and IL-10 production were detected by ELISA. **(D)** The stimulated DCs of each group were collected and cocultured with T cells purified from spleens of BALB/c. DC-T cells were mixed in different proportions for MLR. The proliferation of T cells was determined by Cell Counting Kit-8. Data are presented as the mean ± SD of four independent experiments. *p < 0.05; MHC, major histocompatibility complex; MFI, mean fluorescence intensity; LPS, lipopolysaccharide.

To further explore whether aspirin affects the phenotype and function of DCs, and thus regulates the differentiation of T cells, BMDCs were cultured *in vitro* with or without aspirin, and the phenotypic differences between aspirin-treated and untreated BMDCs were examined. As shown in Figure 4A, the results of flow cytometry analysis implied aspirin exerted no effect on differentiation of DCs from bone marrow cells. However, the expression of MHCII, CD80, CD86, and CD40 molecules on DCs was obviously inhibited (MHCII MFI 20293 ± 1,457 versus 16,787 ± 1,208, *p* < 0.05; CD80 MFI 11968 ± 1771 versus 8,082 ± 286, *p* < 0.05; CD86 MFI 19264 ± 1,481 versus 13,585 ± 2,123, *p* < 0.05; CD40 MFI 5738 ± 870 versus 3,339 ± 327, *p* < 0.05; Figures 4A,B). Subsequently, the cytokines produced in the medium by LPS-stimulated DCs and immature DCs (iDC) were detected by ELISA, and the results indicated that the production of IL-1β and IL-12 of DCs was inhibited in the presence of aspirin, while the IL-10 production was promoted (IL-1β, 415 ± 28.4 versus 107 ± 14.7 pg/ml, *p* < 0.05; IL-12, 572 ± 23.5 versus 317 ± 21.9 pg/ml, *p* < 0.05; IL-10, 48.8 ± 10.4 versus 233 ± 13.7 pg/ml, *p* < 0.05; Figure 4C).

To investigate whether aspirin-treated DCs changed their ability to stimulate T-cell proliferation, the LPS-stimulated BMDCs were generated from bone marrow cells of B6 mice with or without aspirin treatment as the stimulus cells, and T cells were purified from spleens of BALB/c as the response cells for the MLR. The results were shown that aspirin treated DCs significantly weakened the ability of DCs to promote the proliferation of effector T cells (Figure 4D).

### Aspirin Regulates the Maturation of DCs Through the NF-κB Signaling Pathway

**FIGURE 5:**
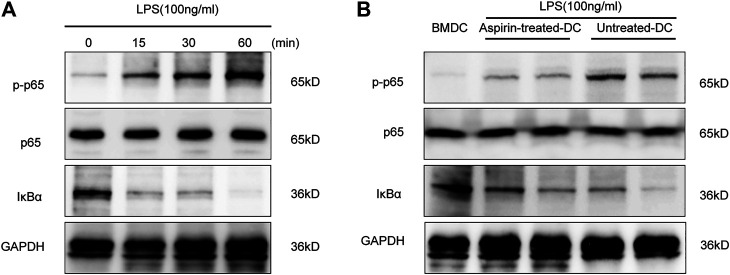
Aspirin regulates the maturation of DCs through the NF-κB signaling pathway. Bone marrow–derived dendritic cells were generated from B6 bone marrow cells with 2 mM aspirin added to the medium, and the DMSO was added only as the untreated control. DCs were collected from each group on the seventh day and administered 100 ng/ml LPS stimulation. **(A)** Western blotting was performed to detect the phosphorylation level of p65 and the degradation level of IκBα of BMDCs under LPS stimulation; GAPDH was used as the internal reference. **(B)** The phosphorylation level of p65 and the degradation level of IκBα in aspirin-treated DCs and untreated DCs under LPS stimulation. GAPDH was used as the internal reference. Data represents four independent experiments. GAPDH, glyceraldehyde-3-phosphate dehydrogenase.

NF-κB activation is an extremely important link with the maturation of DCs (Lim et al., 2018). To ascertain the role of the NF-κB signaling pathway in DC maturation, we detected the phosphorylation level of NF-κB p65 and the degradation level of IκBα by Western blotting in the BMDCs under the stimulation of LPS. As shown in Figure 5A, the phosphorylation level of p65 and the degradation level of IκBα were increased in a time-dependent manner. Therefore, the above experiments were repeated to detect the activity of NF-κB signaling in aspirin-treated DCs and untreated DCs with LPS stimulation. The results indicated that the phosphorylation of p65 and the degradation of IκBα were inhibited in DCs with the aspirin treatment (Figure 5B). Therefore, the aspirin-exerted effect on the maturation of DCs may be accomplished through the NF-κB signaling pathway.

### Aspirin Combined With the Calcineurin Inhibitor Further Antagonizes Cardiac Allograft Rejection

**FIGURE 6:**
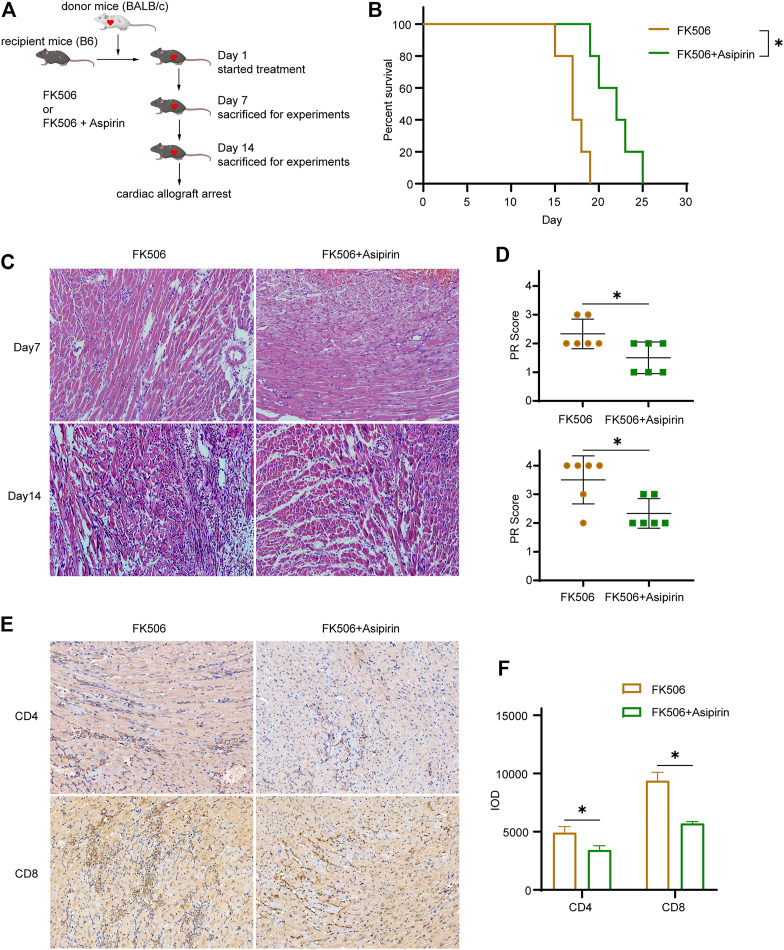
Aspirin combines with calcineurin inhibitor further antagonizes cardiac allograft rejection, establishing the model of allogeneic heart transplantation. From the first day after surgery, FK506 (1 mg/kg/d) or FK506 (1 mg/kg/d) + aspirin (200 mg/kg/day) with an intraperitoneal injection was administered. **(A)** The schematic of treatment and experiment protocols following heart transplantation. **(B)** The combination of FK506 and aspirin treatment significantly prolonged the survival of cardiac allograft (MST, 21.8 ± 1.1 versus 17.2 ± 0.7 days, *n* = 5 per group, *p* < 0.05). **(C)** Cardiac allografts were harvested on 7th and the 14th day after transplantation; the allograft sections were made, and hematoxylin–eosin staining were performed (×40, *n* = 6 per group). The infiltration of inflammatory cells and the necrosis of myocardial cells in allograft were further inhibited in the FK506 + aspirin-treated group compared with the FK506-treated group. **(D)** The PR score was performed on tissue sections of each group on the 7th and the 14th day after transplantation (*n* = 6 per group). **(E**) Cardiac allografts were harvested on the 14th day after transplantation, the tissue sections were made, and immunohistochemical staining of CD4(+) and CD8(+) T cells was performed (×40, *n* = 6 per group). Compared with the FK506-treated group, the infiltration of CD4(+) and CD8(+) T cells in allografts was significantly reduced with the FK506 + aspirin treatment. **(F)** IOD was used to analyze the amount of CD4(+) and CD8(+) T cell infiltration in the allografts in each group (*n* = 6 per group). *p < 0.05; MST, mean survival time; IOD, integrated optical density.

Given that calcineurin inhibitor (CNI) and aspirin inhibit allograft rejection through different mechanisms, we attempted to treat mice with the combination of FK506 and aspirin following heart transplantation (Figure 6A). Compared with FK506 treated alone, FK506 + aspirin treatment significantly prolonged the survival of cardiac allograft (MST, 21.8 ± 1.1 versus 17.2 ± 0.7 days, *p* < 0.05; Figure 6B). According to pathological analysis by hematoxylin–eosin staining, we found that on the 7th and the 14th day after transplantation, the infiltration of inflammatory cells in cardiac allograft and the degree of myocardial cells necrosis were obviously reduced with the combined treatment (Figure 6C). Correspondingly, the PR score of the cardiac allograft in the FK506 + aspirin-treated group was also lower than that of the cardiac allograft in the FK506-treated group (Day 7 2.33 ± 0.52 versus 1.50 ± 0.55, *p* < 0.05; Day 14 3.50 ± 0.84 versus 2.33 ± 0.52, *p* < 0.05; Figure 6D). We simultaneously analyzed the infiltrated T cells in the cardiac allograft by immunohistochemistry, and the results suggested that FK506 combined with aspirin treatment had a more inhibitory effect on the infiltration of CD4 (+) T cells and CD8 (+) T cells in allograft (CD4 IOD 4920 ± 525 versus 3,421 ± 368, *p* < 0.05; CD8 IOD 9376 ± 724 versus 5,730 ± 165, *p* < 0.05; Figures 6E,F).

## Discussion

With the expansion of pharmacological studies, the survival rate of patients following transplantation has been considerably improved. However, the immunosuppressive agents have several side effects, such as cancer, infection, diabetes, and cardiovascular disease (Ye et al., 2017). Therefore, there is an urgent need to find the effective treatment regimen with fewer side effects to replace or reduce the use of immunosuppressants. In this study, we have demonstrated that aspirin reduced the expression of co-stimulatory molecules on DCs through the NF-κB signaling pathway and inhibited the proliferation and differentiation of T cells, which effectively prolonged the allograft survival after heart transplantation. In addition, we also found that the synergistic effect of combining CNI and aspirin could further alleviate the cardiac allograft rejection.

Aspirin is the first nonsteroidal anti-inflammatory drug to be discovered. Recent studies have found that aspirin played a role in many other diseases, such as tumors (Hua et al., 2019; Lichtenberger and Vijayan, 2019), preeclampsia (Atallah et al., 2017), Alzheimer’s disease (Berk et al., 2013; Wang et al., 2015), and sepsis (Leijte et al., 2019). Additionally, immunologists discovered that aspirin exerted a regulatory effect (non–COX-dependent) on the immune system. Regarding autoimmune diseases, aspirin had a good therapeutic effect on rheumatoid arthritis and systemic lupus erythematosus (Yamazaki et al., 2002; Zhang et al., 2007). Concerning organ transplantation, a meta-analysis study reported that administration of aspirin following kidney transplantation protected allograft function (Cheungpasitporn et al., 2017). Moreover, a lot of clinical studies have shown that aspirin successfully reduced the risk of allograft failure in heart transplant patients and prolonged the survival of allograft (Kim et al., 2017; Peled et al., 2017). However, the underlying mechanism of aspirin is elusive. In this study, we used the murine model of heterotopic heart transplantation to demonstrate that aspirin could effectively prolong the allograft survival in mice. Pathological analysis and PR score of cardiac allografts indicated that aspirin treatment had a meaningfully protective effect on acute rejection after transplantation. During organ transplantation, T cells play an important role in the process of allograft rejection. The CD4(+) T cells, through the direct and indirect manners, recognize allo-antigens and initiate immune response. The CD8(+) T cells, with the auxiliaries of APC MHCI molecules or (and) CD4(+) T cells, differentiate into cytotoxic T lymphocytes (CTLs) and directly damage the allograft (Brummelman et al., 2018; Siu et al., 2018). In our study, we found that the proportion of CD8(+) T cells decreased in the spleen of recipient mice treated with aspirin, which may closely associate with the inhibition of acute cardiac allograft rejection. However, CD4(+) T cells were not affected by aspirin. The CD4(+) T helper cells include Th1, Th17, Th2, and regulatory T cells. Th1 cells initiate acute cellular rejection primarily by producing IFN-γ and IL-2; Th17 cells produce IL-17 and other pro-inflammatory cytokines to mediate acute and chronic rejection; Th2 cells secrete IL-4, IL-5, and IL-12, which are considered to be involved in chronic allograft rejection (Koutsokera et al., 2018). In contrast, Treg cells play an important role in immune regulation and tolerance (Romano et al., 2019). According to our study on the seventh day after transplantation, results of real-time PCR indicated that Th1 and Th17 cells decreased and Treg cells increased in the cardiac allograft after aspirin treatment. However, the differentiation of Th2 cells was not affected. Flow cytometry analysis revealed that aspirin inhibited the differentiation of IFN-γ(+)CD4(+) T cells and IFN-γ(+)CD8(+) T cells as well as induced Foxp3(+)CD4(+) Treg cells in the spleen. In addition, we found that while the IL-17 transcription in cardiac allograft was inhibited by aspirin, it had no effect on IL-17(+)CD4(+) T cells in the spleen. This phenomenon may result from several factors, including the chemokine production in cardiac allograft. Therefore, the present results bear out that aspirin inhibits the pro-inflammatory cells and promotes the anti-inflammatory cells in mice, in favor of reducing acute cardiac allograft rejection.

DCs play an important role in the initiation of transplant-related innate and adaptive immune response. They are crucial for the activation of allogenic reactive T cells and indispensable for the regulation of immune response (Tiao et al., 2005). Following organ transplantation, the activation state and maturation degree of DCs determined the immune response or immune regulation to the allograft (Takenaka and Quintana, 2017). Immature DCs process and present antigens to T cells; however, due to the lack of sufficient co-stimulatory signals, allogenic reactive T cells will not proliferate and activate effectively, eventually leading to immune hyporesponsiveness or tolerance. In contrast, mature DCs can adequately stimulate T cells and orchestrate antigen-specific immune responses (Morelli and Thomson, 2007). The maturation of DCs is marked by the increased expression of surface molecules which is involved in stimulating T-cell proliferation, such as MHCII, CD80, CD86, and CD40 (Tan and O'Neill, 2005). In the current study, we found that aspirin did not affect the differentiation of DCs *in vitro*; however, it can clearly decrease the expression of co-stimulatory molecules such as CD80, CD86, CD40, and MHCII on DCs. Meanwhile, the pro-inflammatory cytokines (IL-12 and IL-1β) were inhibited, and the anti-inflammatory cytokine (IL-10) was promoted in DCs with the aspirin treatment. *In vivo*, we also found that the expression of co-stimulatory molecules such as CD80, CD86, and CD40 on DCs was downregulated as well as the proportion of DCs decreased in the spleen after aspirin treatment. Interestingly, aspirin did not suppress the expression of MHCII on DCs in mice, which may provide a favorable condition for the antigen-specific tolerance. The process that antigens being presented to CD4 (+) T cells by MHCII is the prerequisite for immune tolerance (Jurewicz and Stern, 2019). In the immune system, MHCII molecules of DCs present the antigen peptide to the T cells, which is called the first signal. The co-stimulatory molecules on DCs provide the second signal and the cytokines provide the third signal to the T cells, which determines the outcome of the immune response and tolerance. The different effects of aspirin exerted on the MHCII between *in vitro* and *in vivo* may result from multiple factors including the origin and microenvironment of dendritic cells as well as the pharmacokinetics and pharmacodynamics of aspirin. Moreover, in the results of MLR, we found that the capacity of aspirin-treated DCs in promoting T-cell proliferation was strikingly diminished. After reviewing our current study data, we may be able to make the tentative determination that aspirin can inhibit the maturation and alter the function of DCs, thus induce the immune hyporesponsiveness of effector T cells or immune tolerance.

Toll-like receptor–mediated activation of NF-κB has been reported to be involved in the maturation of DCs (Li et al., 2017). In our study, we found that NF-κB activation was inhibited in aspirin-treated BMDCs in the presence of LPS stimulation. Therefore, we hypothesized that aspirin may inhibit the maturation of DCs through manipulating the activation of the NF-κB signaling pathway. Calcineurin inhibitor, such as FK506, prevents graft rejection by inhibiting T-cell proliferation, which has a different mechanism from aspirin in protecting allograft rejection (Juvvadi et al., 2019). Therefore, to further determine the application value of aspirin in therapy of allograft rejection, we combined aspirin with FK506 to treat heart transplanted mice. The results showed that aspirin synergized with FK506 could effectively improve the therapeutic effect for allograft rejection, which suggests that combining aspirin with the existing T-cell immunosuppressants may be a better therapeutic regimen for transplant patients in clinical practice.

However, there are some limitations in our study: ①our study only revealed that aspirin inhibited acute rejection, and whether it plays a protective role in chronic rejection remains unknown; ②the direct effect of aspirin on other immune cells, such as T cells, macrophages, neutrophils, and NK cells, is not fully understood and needs to be studied further; ③the best dose of aspirin and the optimal administration mode (gavage or injection) for cardiac allograft rejection inhibition need to be further explored; ④whether aspirin protects against other solid organ (skin, liver, kidney, *etc*.) rejection in mice is unclear; ⑤whether the side effects (gastrointestinal reactions, bleeding, *etc*.) of long-term aspirin treatment are detrimental to the recipient’s survival should be carefully determined.

In conclusion, this study demonstrates the efficacy of aspirin on alleviating acute cardiac rejection in mice. Aspirin inhibits the maturation of DCs by affecting the NF-κB signaling pathway, induces the immune hyporesponsiveness of effector T cells, and then attenuates the acute cardiac allograft rejection following heart transplantation. This finding may provide a new direction and pharmacological basis for optimizing the immunosuppressive regimens in anti-rejection treatment.

## Data Availability

The raw data supporting the conclusions of this article will be made available by the authors, without undue reservation.
